# Genetics of Alzheimer’s disease

**DOI:** 10.1007/s10354-021-00819-9

**Published:** 2021-02-22

**Authors:** Theresa König, Elisabeth Stögmann

**Affiliations:** Department of Neurology, Medical University of Vienna, Vienna, Austria

**Keywords:** Dementia, Neurodegeneration, Neurogenetics, Genetic profiling, Risk variants

## Abstract

Alzheimer’s disease (AD) is the leading cause of neurodegeneration in the elderly and is clinically characterized by slowly progressing cognitive decline, which most commonly affects episodic memory function. This eventually leads to difficulties in activities of daily living. Biomarker studies show that the underlying pathology of AD begins 20 years before clinical symptoms. This results in the need to define specific targets and preclinical stages in order to address the problems of this disease at an earlier point in time. Genetic studies are indispensable for gaining insight into the etiology of neurodegenerative diseases and can play a major role in the early definition of the individual disease risk. This review provides an overview of the currently known genetic features of AD.

## Introduction

Alzheimer’s disease (AD) is a neurodegenerative disease, neuropathologically characterized by the deposition of misfolded proteins. Those are amyloid plaques on the one hand and tau tangles on the other. Another important neuropathological correlate is neurodegeneration, which is topographically associated with tau pathology and characterized macroscopically by cerebral atrophy and microscopically by the loss of neurons. Regarding cognitive dysfunction, clinical and neuropathological studies show a much closer association with neurofibrillary tangles (insoluble tangled fibers consisting primarily of tau) than with amyloid plaques. Generally, the closest association with cognitive dysfunction was shown for neurodegeneration, in particular with loss of synapses [1]. The most common neuropathological finding in patients with clinically and neuropathologically diagnosed AD is a mixed pathology. Thus, in addition to the above-mentioned changes, pathologies such as cerebrovascular disease and deposition of other proteins such as TAR DNA-binding protein 43 (TDP-43) and synuclein, or abnormal structures like Lewy bodies occur. The appearance of several coexisting pathologies seems to be inevitably linked to ageing.

For early diagnosis and better monitoring of such a heterogeneous disease, in vivo biomarkers are particularly useful. Knowing that the underlying pathology starts up to 20 years before the first clinical symptoms of AD manifest, this presumably long therapeutic window could be used to enable an early and better stratification of diagnosis and potential therapy of patients.

## Relevance for clinics

Heritability—the proportion of phenotypic variance that can be explained by genetic factors—was reported to be 60–80% for the entire spectrum of AD [2]. A more recent study from John Hardy and colleagues based on a polygenic score predicts a heritability of 84% for the risk of AD, which is in concordance with previous works [3].

In the late-onset form of AD (so-called sporadic form with onset after the age of 65, LOAD), which applies to 95% of cases, it is assumed that the underlying etiology is caused by a combination of genetic components and environmental factors in a ratio of about 70:30, respectively [4]. In the few cases of early onset AD (EOAD), the etiology of the disease is likely to be substantially or even almost exclusively genetic, even though not all patients have a positive family history (up to 60%) and only few patients of those familial EOAD cases show a clear autosomal dominant mode of inheritance (10–15%) [5–7]. A study with a large cohort of patients with probable AD cases over 5 years showed a heritability of 92–100% for EOAD, based on the concordance of disease in the family and the prevalence of the disease in the population [8].

Thus, genetics seem to play a major role in all forms of AD. Nevertheless, a clear distinction must be made between patients with a monogenic and those with a complex mode of inheritance.

A potential clinical benefit of deciphering the genetic background is the stratification into high- and low-risk groups based on different genetic variants. This risk stratification could in turn become relevant for future prevention studies.

## Genetics of complex diseases

**Fig. 1:**
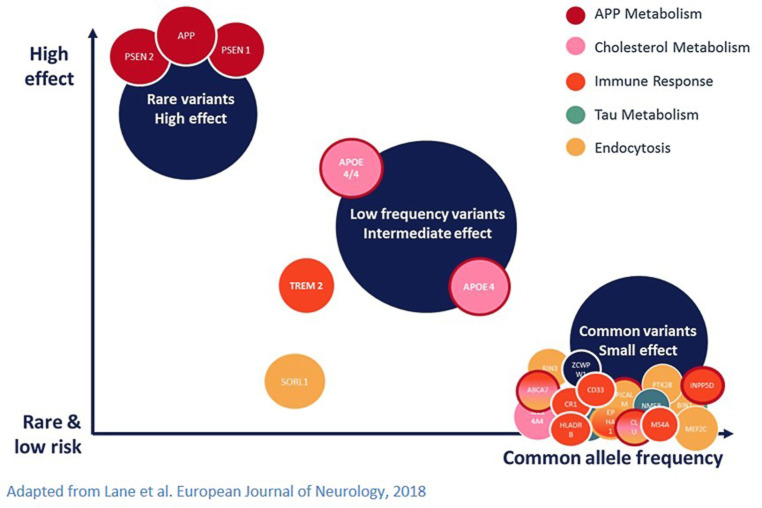
Risk genes associated with AD. Previously identified genetic variants, represented by the frequency of risk alleles and the strength of the genetic effect. Colors in the legend indicate pathways in which the genes are involved. (Adapted from Lane et al. European Journal of Neurology, 2018 Risk genes associated with AD)

The genetics of complex diseases can be best explained by the model of Manolio et al., which was adapted for AD from Lane et al. ([9, 10]; Fig. 1). Here, the significance of a genetic variant is quantified on the basis of frequency on the one hand and effect of the variant on the other hand. As illustrated, high penetrant alleles have a rather low allele frequency in the population, whereas low susceptibility alleles such as variants found in genome-wide association studies (GWAS) are common in the population.

## Monogenic AD forms

A very small proportion of patients with EOAD shows Mendelian inheritance. Carriers of mutations in certain genes, namely amyloid precursor protein (*APP*), presenilin 1 (*PSEN1*) and presenilin 2 (*PSEN2*), are almost guaranteed to develop AD [11–17] and these mutations can be regarded as the actual cause of the disease. The inheritance pathway is autosomal dominant, which means that one mutation in one of the two parental gene copies (alleles) is sufficient to cause the disease. Autosomal dominant inheritance is typically characterized by a “vertical” inheritance pathway, i.e., the disease is inherited over generations. Children of affected individuals have a 50% risk of inheriting the mutated allele and passing it on themselves.

APP is a type 1 transmembrane protein that is predominantly cut by the protease α‑secretase producing nonpathogenic soluble fragments [18]. Alternatively, the extracellular part of APP can be cleaved by the β‑secretase, also called BACE1 (β-site of APP cleaving enzyme) [19]. The resulting extracellular fragment is soluble, while the remaining transmembrane/intracellular part of APP is further cleaved by the γ‑secretase. Notably, PS‑1 and PS‑2 (encoded by *PSEN1* and *PSEN2*) are parts of the γ‑secretase protein complex [20]. During this second cleavage, the 36–43 amino acid long β‑amyloid peptide (Aβ) is generated and released into the cytoplasm. Aβ40 and 42 are particularly prone to aggregation into toxic oligomers and lead to the formation of amyloid plaques [21–23]. Mutations in *APP, PSEN1* or *PSEN2* lead to overproduction of pathological Aβ fragments and consequently to amyloid pathology with increased plaque formation [24].

In the small group of monogenic AD patients, the well-researched underlying pathophysiological process suggests a simple amyloid proteinopathy and addresses it as a specific target. The Dominantly Inherited Alzheimer Network (DIAN) is an international registry of autosomal dominant AD families. DIAN performs long-term monitoring of symptomatic and asymptomatic *APP, PSEN1* and *PSEN2 *carriers including evaluation of biomarkers in the cerebrospinal fluid (CSF) and regular performance of magnetic resonance imaging (MRI), positron emission tomography (PET) and neuropsychological tests [25–27]. At the same time, symptomatic and asymptomatic mutation carriers can optionally participate in a clinical study with anti-Aβ antibodies [28].

The Alzheimer Prevention Initiative (API) is pursuing a similar approach [29]. One study arm is investigating the chronological sequence of diagnostic biomarkers and the efficacy of another anti-Aβ antibody (crenezumab) in the world’s largest known autosomal dominant AD family tree with about 1500 *PSEN1* mutation carriers in Colombia.

## Multifactorial AD forms

The large proportion of LOAD with a disease onset after the age of 65 has a multifactorial etiology. Although heritability is also estimated to be high for LOAD, no clear Mendelian pathway can be established in these patients. It is assumed that complex genetic interactions, or gene–environment interactions, contribute to the development of the disease [30]. Patients with multifactorial AD forms can be further divided into carriers and noncarriers of the apolipoprotein E (*APOE*) risk allele (*APOE4*). These two groups are discussed in more detail below.

### Patients carrying one or two *APOE4* alleles

The by far largest single genetic risk factor for LOAD is apolipoprotein E (*APOE*), which has been known since 1993. There are three common isoforms of *APOE* (alleles *APOE2, APOE3* and *APOE4*), which result from polymorphic variation in the gene. *APOE4* is associated with higher risk of AD [31]. However, *APOE4* cannot be regarded as causal in the development of AD, since it is neither sufficient nor necessary to cause AD. This means, individuals carrying one or two of the *APOE4* risk alleles will not certainly develop AD and individuals without *APOE4 *are not protected. However, its high importance can be explained by its relatively high frequency in the population combined with a relatively high effect strength, which is also illustrated in Fig. 1. Other known genetic variants either occur many times less frequently or have only minor effect on disease development.

The alleles *APOE2, APOE3* and *APOE4* have a frequency of 8.4%, 77.9% and 13.7%, respectively, in the normal population worldwide. Lifetime risk for the most frequent genotype *APOE* 3/3 (i.e., both maternal and paternal alleles are *APOE3* alleles) is about 10–15% [32]. The risk of AD increases in individuals with genotype *APOE* 2/4 (one allele is an *APOE2* allele, the second allele is an *APOE4* allele) with an odds ratio (OR) of 2.6, with an *APOE* 3/4 genotype with an OR of 3.2 and with an *APOE* 4/4 genotype with an OR of 14.9 [33, 34]. The risk to develop AD for *APOE4* homozygotes is estimated to be about  40–50% at the age of 70 or 85 [32, 35]. Conversely, the *APOE2* allele has a putative protective effect against the development of AD. The risk of AD in individuals with an *APOE* 2/2 or *APOE* 2/3 genotype decreases with an OR of 0.6 compared to individuals with an *APOE* 3/3 genotype [36]. In addition, the presence of one or two* APOE4* alleles leads to an earlier onset of disease. The average age of clinical onset is 68 years in *APOE4*-homozygous patients, 76 years in *APOE4*-heterozygous patients and 84 years in *APOE4*-negative patients [37].

The *APOE4* allele has been described to promote amyloid deposition starting already in middle age. PET studies in cognitively healthy individuals in various age groups have shown that *APOE4*-positive individuals exhibit amyloid deposition significantly earlier than *APOE4*-negative individuals [16]. Although *APOE4* has been known as a risk factor for a very long time, there remains disagreement over the specific mechanisms by which the *APOE4* allele increases the risk of AD and age-related cognitive decline.

The protein ApoE, among other functions, acts as a ligand for the low-density lipoprotein receptor (LDL-receptor) and the very low-density lipoprotein receptor (VLDL-receptor) and is strongly expressed in the brain, especially in astrocytes. ApoE-containing lipoproteins, which are secreted by glial cells, bind to these lipoprotein receptors and are taken up into the neurons. ApoE is the main transport protein for extracellular cholesterol and other lipids and mediates the lipid exchange between neuronal and non-neuronal cells [38]. A relation between the *APOE4* allele and multiple pathological impacts, including on amyloid deposition, synaptogenesis, mitochondrial function and phosphorylation of tau, has been suggested [39, 40].

**Fig. 2:**
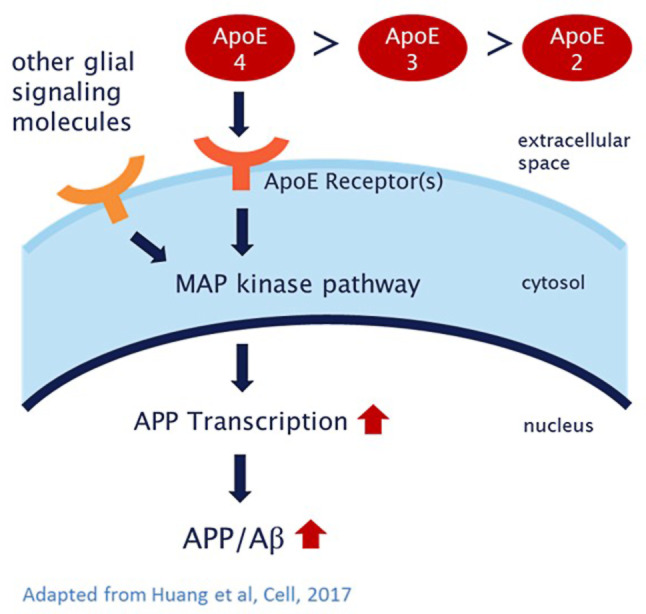
Simplified, schematic representation of the ApoE signaling pathway, which controls *APP* transcription and Aβ production by activating a MAP kinase cascade. ApoE increases the risk of AD by causing a gradual increase in APP abundance and Aβ secretion, with ApoE4 being more and ApoE2 less efficient than ApoE3, in parallel with its effects on AD risk. (Adapted from Huang et al., Cell, 2017 Stimulation of APP transcription and Aβ secretion by ApoE)

A study led by Thomas Südhof at Stanford University [41] provides a possible new comprehensive and coherent explanation on how the *APOE *genotype influences the risk of developing AD. In this work they employed a human neuronal cell culture system to show that ApoE secreted by glia stimulates neuronal β‑amyloid production. It seems that ApoE, by binding to a non-canonical ApoE receptor, activates a MAP kinase pathway leading to an increase in *APP* transcription, which in turn leads to an increased production of Aβ. ApoE4 has been shown to activate the pathway more strongly than ApoE3 or ApoE2. If this proposed mechanism holds to be true, it would be a further piece of evidence for the amyloid hypothesis, insofar as the most important risk factor, *APOE4* confers its pathogenic effect by increasing β‑amyloid. This could lead to a cumulative effect in people with *APOE4* alleles during their lifetime ([42]; Fig. 2).

*APOE4* is also thought to influence tau pathology in AD. In mice carrying transgenic human pathogenic tau mutations and human *APOE4* variants, significantly higher tau levels and a more pronounced neurodegeneration in the brain were found. Furthermore, these mice also showed increased neuroinflammation compared to controls [43].

### APOE4 noncarriers

A large proportion of AD patients cannot be etiologically attributed to pathogenic *APP, PSEN1, PSEN2* variants or *APOE4* genotype.

In GWAS, so-called common variants (occurrence of the rare allele in the population > 5%) with rather low effects are detected. Here, associations of loci (positions on the DNA that are not necessarily within the coding region code for an entire gene) are detected in many thousands of patients and their occurrence is compared between patient cases and controls. This means, the associated SNPs mark a region of the human genome that may influence the risk of disease. Although these studies cannot establish a causality between genes and disease, they have provided important insights into basic pathophysiological processes of AD, which have been validated in numerous subsequent studies. The most recent meta-analysis, which was published in 2019, examined about 90,000 cases of sporadic AD. The loci found were named after the gene in the closest possible proximity. The main pathophysiological pathways were associated with inflammation, APP processing and lipid metabolism. Tau processing and endocytic processes may also play a minor role [44].

Further insights have been gained in recent years with next-generation sequencing (NGS). In NGS studies, thousands of patients are examined for so-called rare variants (occurrence of the rare allele in the population < 2%), which in turn have a slightly higher effect strength. Targeted gene sequencing panels are useful tools for analyzing specific mutations in a particular cohort. These panels contain a selection of genes or gene regions that have known or suspected associations with the disease or phenotype under investigation. One of the most interesting genes discovered here is *TREM2* (triggering receptor expressed on myeloid cells 2). *TREM2* was originally discovered in the rare recessive disorder Nasu-Hakola, in which homozygous loss-of-function mutations lead to a severe form of dementia as well as bone cystic lesions [45, 46]. In a large rare variant association analysis, it was discovered that some heterozygous variants in *TREM2* increase susceptibility for AD with ORs of about 2–5. Particularly one risk variant, R47H (rs75932628) was discovered to be very common with a frequency of about 0.005 in the Caucasian population (1 out of 200 individuals is a carrier of this variant, OR 2.92–4.59) [47, 48]. TREM2 is known to modulate microglia activity [47]. However, it remains unclear, how variants in the gene exactly contribute to AD. Thus, understanding the role of *TREM2* might give valuable insight into neuroinflammatory mechanisms in this disease [49].

In addition, an association between mutations in the DNA demethylase ten-eleven translocation 2 (*TET2*) and elevated risks for Alzheimer’s disease, frontotemporal dementia, and amyotrophic lateral sclerosis was recently reported [50] with combined analysis OR of 2.3–3.7. In mouse models and brains of AD patients, TET2 was shown to be elevated in microglia, particularly those surrounding amyloid plaques. Generally, TET2 is suggested to promote a proinflammatory response in microglia [51].

Variants discovered in NGS studies, such as those mentioned above, are much more common compared to those found in *APP, PSEN1* and *PSEN2* and rarer than *APOE4*. Compared to the ORs of variants found by GWAS, however, their effect size is much higher.

## Microglia in AD

Microglia are the resident phagocytes of the CNS and continuously monitor the CNS with their cell protrusions. In addition, they play a role in the plasticity of neuronal connections and contribute to modelling of synapses. Among many other stimuli, pathological Aβ deposits lead to the activation of microglia inducing various cellular changes. This includes morphological changes, changes in surface marker expression and secretory profile with increased proliferative responses as well as the release of proinflammatory cytokines such as TNF‑α and interleukin-1β [52]. Microglia take up and degrade soluble Aβ oligomers and Aβ fibrils via pattern recognition receptors (PRRs) such as receptors for advanced glycation end products (RAGE) [53], toll-like receptors (TLRs) [54] and scavenger receptors [55, 56]. In principle, the acute inflammatory processes contribute to the removal of Aβ and to the homeostasis of the CNS. However, repeated activating stimuli can trigger a phenomenon called priming rendering microglia more sensitive and resulting into exacerbated inflammatory responses upon further re-activation [57]. Priming in AD is presumably mediated by various mechanisms—on the one hand probably by the permanent presence of pathological Aβ, on the other hand also by accelerated microglial activation related to the aging brain [58]. Factors such as systemic inflammation are also likely to play a reinforcing role. It has been shown that interleukin-1β enhances plaque formation by modulating APP expression [59]. Furthermore, upregulation of the enzyme BACE1 by cytokines is likely to lead to increased production of Aβ species [60].

## Use of risk scores

The current findings in genetic research help us to move towards more personalized medicine. Several studies already investigated the importance of genetic markers for the prediction of AD risk. In a recent work an estimate of the risk of developing AD was calculated based on genotype and age. This polygenic hazard score (PHS) is the sum of the weighted age-specific AD risk compared to the general population. The score is based on single nucleotide polymorphisms (SNPs) at 31 sites in the genome, which have previously been detected in multiple GWAS analyses. A significantly higher and earlier risk of AD could be predicted for individuals in the highest PHS percentiles [61]. Genetic variants are integrated into an epidemiological framework [62]. PHS correlates not only with the extent of amyloid and tau accumulation and cortical degeneration, but also with the loss of cognitive abilities during progression of disease. This score could therefore be a useful screening tool in the future [63].

Interestingly, a recent study reports that PHS for AD, when calculated for men and women separately, show sex-specific differences [64]. The authors showed that sex-matched scores lead to improved prediction of disease onset, progression, and neuropathology in comparison to scores calculated for all study participants together, while there was no difference in assessing prevalence. The precision of polygenic risk scores in predicting AD was the same in men and women. It is elusive which gene variants are involved in this sex difference and answering this question will require further studies.

## Missing heritability

Despite major advances in recent research regarding the genetic background of AD, all known genetic factors taken together explain less than half of the heritability of the disease. This fact is true for many genetically complex diseases [2, 65]. The reason for this “missing heritability” can only be guessed at the present. Answers are likely to lie in families with rare or even private genes with reduced penetrance not captured by current genotyping platforms and in common variants with small effect sizes [66–68]. The analysis of rare variants is much more difficult and less powerful than that of frequent variants. It requires a large sample size to reliably detect a rare variant. As shown, it takes at least 460 and 4600 individuals, respectively, to detect alleles with a frequency of 0.5% or 0.05% with a probability of 99% [69]. Furthermore, more stringent significance levels are required, since the number of rare variants exceeds the number of frequent variants by a multiple, thus, resulting in reduced power.

Another aspect of the peculiarities of rare variants can be seen in the example of an interesting *APP* variant (A673T). This missense mutation was found at an APP binding site of β‑secretase and was associated with a 40% reduction of Aβ40 and Aβ41 levels. This variant is thought to have a protective function in carriers, as it is associated with reduced amyloid deposition and a 5-fold lower risk of AD. It also showed a positive effect on the reduction of Aβ in older healthy individuals [70]. Notably, this *APP* variant is extremely rare (0.13% in AD cases and 0.45 to 0.79% in controls in the Icelandic population, 0.011% in AD cases and 0.018% in controls in the American population) but confers a large protective effect on carriers. It was confirmed in Scandinavian countries, but could not be found in North America and Southeast Asia [71–74], limiting its relevance. Nevertheless, the identification of such protective variants leads to a valuable increase in knowledge and contributes to the development of therapeutic strategies.

The phenomenon of missing heritability can be further explained by the existence of many structural variants, gene–environment interactions, parent-of-origin effects, or inflated heritability estimates [75–78]. In addition, epistatic interactions have to be considered. Genes are constantly interacting, which is crucial, for example, for gene regulation, signal transduction and biochemical networks [77]. Epistasis measures the interactive effects between a gene or variant and one or more other genes or variants. Thus, if a gene locus is solely viewed as a self-contained unit without considering its potential interactions, its influence on disease can be overlooked [79].

## Genomic mosaics

Somatic changes in individual cells lead to a variability of unique genomes in a single person and this genetic mosaicism increases during ageing.

A remarkable mechanism had recently been described, which could possibly play a major role in the development of sporadic AD. As shown in 2015, individual neurons in the cortex of AD patients contain more DNA, so-called DNA content variations, leading to increased copy numbers of the *APP* gene [80]. This raises the question whether the number of *APP* genes is also increased in the sense of genomic mosaics in the brain of AD patients. A recently published paper offers an answer and explanation to this question. The work describes a DNA recombination process in which mRNA from somatic cells is reverse transcribed into complementary DNA (cDNA), which is then inserted at random positions in the genome (e.g., in strand breaks of the DNA) as so-called genomic cDNA (gencDNA). Somewhere along the way between mRNA and cDNA insertion,* APP *exons are lost, point mutations occur and different insertions and deletions are introduced, resulting in thousands of *APP* variants in a single brain [41]. The number of* APP* copies is thus increased on the one hand, but on the other hand these copies are also defective. Some gencDNA variants, including pathogenic *APP* mutations which are known from familial AD cases, lead to toxic proteins, resulting in cell death. This would represent a potentially new disease mechanism contributing to the development of sporadic AD.

However, this theory is currently debated in the field and more studies are needed to confirm this mechanism.

## Summary

Since the onset of pathological alterations in AD patients occurs up to 20 years prior to the clinical onset, disease-modifying therapies should be applied as soon as possible in order to be able to achieve the desired beneficial results. Thus, there is an increasing urgency to define preclinical stages and specific targets in order to enable better stratification of patients and to ensure early and differentiated diagnosis and treatment.

Genetic profiling will therefore certainly be an indispensable tool in the future to gain insight into the etiology of complex neurodegenerative diseases and to define individual risk at an early stage. In a very small selected patient population, genetics is already being used to carry out early and hopefully targeted therapies in the course of clinical studies. The final results of these studies and further insight into the genetics of AD are eagerly awaited.
